# MUC1-C confers EMT and KRAS independence in mutant KRAS lung cancer cells

**DOI:** 10.18632/oncotarget.2360

**Published:** 2014-08-22

**Authors:** Akriti Kharbanda, Hasan Rajabi, Caining Jin, Maroof Alam, Kwok-Kin Wong, Donald Kufe

**Affiliations:** Dana-Farber Cancer Institute, Harvard Medical School, Boston, MA 02215

**Keywords:** KRAS, NSCLC, MUC1-C, AKT, ZEB1, EMT, self-renewal

## Abstract

Non-small cell lung cancers (NSCLCs) that harbor an oncogenic KRAS mutation are often associated with resistance to targeted therapies. The MUC1-C transmembrane protein is aberrantly overexpressed in NSCLCs and confers a poor outcome; however, the functional role for MUC1-C in mutant KRAS NSCLC cells has remained unclear. The present studies demonstrate that silencing MUC1-C in A549/KRAS(G12S) and H460/KRAS(Q61H) NSCLC cells is associated with downregulation of AKT signaling and inhibition of growth. Overexpression of a MUC1-C(CQC→AQA) mutant, which inhibits MUC1-C homodimerization and function, suppressed both AKT and MEK activation. Moreover, treatment with GO-203, an inhibitor of MUC1-C homodimerization, blocked AKT and MEK signaling and decreased cell survival. The results further demonstrate that targeting MUC1-C suppresses expression of the ZEB1 transcriptional repressor by an AKT-mediated mechanism, and in turn induces miR-200c. In concert with these effects on the ZEB1/miR-200c regulatory loop, targeting MUC1-C was associated with reversal of the epithelial-mesenchymal transition (EMT) and inhibition of self-renewal capacity. Loss of MUC1-C function also attenuated KRAS independence and inhibited growth of KRAS mutant NSCLC cells as tumors in mice. These findings support a model in which targeting MUC1-C inhibits mutant KRAS signaling in NSCLC cells and thereby reverses the EMT phenotype and decreases self-renewal.

## INTRODUCTION

Approximately 25% of patients with non-small cell lung cancer (NSCLC) harbor an oncogenic *KRAS* mutation that is often associated with resistance to conventional and targeted therapies [1]. NSCLC cells expressing activated KRAS are therefore potential targets for KRAS inhibitors. However, pharmacologic inhibition of mutant KRAS has not as yet proven successful, a situation that has necessitated a focus on therapeutic approaches using inhibitors of the downstream AKT and MEK pathways. In this context, concurrent inhibition of AKT and MEK signaling has been shown to be effective in inducing regressions of mutant *Kras*-driven murine lung adenocarcinomas [2] and this strategy is being evaluated for the treatment of patients with mutant KRAS NSCLC. Other potential targets for inhibiting growth of mutant KRAS NSCLC cells have been identified in synthetic lethal RNAi and drug screens. For example, the non-canonical IκB kinase TBK1 and downstream NF-κB signals are essential for survival of mutant KRAS NSCLC cells [3]. GATA2 and CDK4 have also been shown to be of importance for the growth and survival of NSCLC cells expressing mutant KRAS [4, 5]. In a small molecule screen, mutant KRAS NSCLC cells were more sensitive to inhibition of the RAF→MEK→ERK pathway as compared to KRAS wild-type cells [6]. By contrast, such selectivity for KRAS mutant cells was not observed with inhibitors of the PI3K→AKT→mTOR pathway [6]. Notably, not all NSCLC cells expressing mutant KRAS are dependent on KRAS for survival [7]. In this context, KRAS-dependent NSCLC cells exhibit a well-differentiated phenotype, whereas KRAS-independent cells are associated with the epithelial-mesenchymal transition (EMT) [7]. Treatment of KRAS-dependent NSCLC cells with TGFβ1, an inducer of EMT, reduces dependence on KRAS, further indicating that EMT contributes to KRAS independence [7]. These findings and the demonstration that sensitivity of NSCLC cells to EGFR inhibitors is inhibited by EMT [8] have supported an association between EMT and loss of oncogene addiction.

Mucin 1 (MUC1) is a transmembrane heterodimeric protein that is aberrantly expressed in NSCLCs. Over 80% of NSCLCs of the adenocarcinoma subtype express MUC1 at high levels [9]. In addition, the overexpression of MUC1 in NSCLC is associated with poor disease-free and overall survival [9–13]. Of importance to understanding its function in NSCLC, MUC1 is translated as a single polypeptide that undergoes autocleavage into two subunits that, in turn, form a stable non-covalent heterodimer at the cell surface [14]. The MUC1 N-terminal subunit (MUC1-N) contains glycosylated tandem repeats that are characteristic of the mucin family. The MUC1 C-terminal subunit (MUC1-C) is a single-pass transmembrane protein that interacts with receptor tyrosine kinases, such as EGFR and others [14]. Moreover, the MUC1-C 72 amino acid cytoplasmic tail contains multiple phosphorylation sites and interacts with diverse effectors that have been linked to transformation [14]. For example, MUC1-C contributes to activation of the canonical NF-κB pathway by constitutively interacting with the IκB kinase (IKK) complex in cancer cells and in the response to inflammatory cytokine stimulation of non-malignant epithelial cells [15]. MUC1-C also binds directly to NF-κB p65 and promotes NF-κB-mediated gene transcription [16]. The available evidence in breast cancer cells indicates that involvement of MUC1-C in NF-κB signaling is linked to the induction of EMT and self-renewal [17, 18]. In this way, MUC1-C occupies the ZEB1 promoter with NF-κB and thereby promotes ZEB1 transcription [17]. In turn, MUC1-C associates with ZEB1 and the MUC1-C/ZEB1 complex suppresses transcription of miR-200c, an inducer of epithelial differentiation [17]. MUC1-C also induces breast cancer cell sphere formation, a characteristic that is associated with EMT and self-renewing stem cells, by an NF-κB-dependent mechanism [18]. To our knowledge, there is nothing known about involvement of MUC1-C in EMT or self-renewal of NSCLC cells. In addition, there have been no reports linking MUC1-C to KRAS addiction in lung or other cancer cells.

The overexpression of MUC1 in NSCLCs and other types of carcinomas has supported the attractiveness of MUC1-N and MUC1-C as potential cancer targets [19]. In this context, underglycosylation of the MUC1-N tandem repeats in tumor cells, as compared to normal epithelia, provided the basis for targeting MUC1-N with antibodies and vaccines [14]. Additionally, the oncogenic MUC1-C subunit contains a CQC motif in the cytoplasmic domain that is necessary and sufficient for MUC1-C homodimerization and function [20, 21]. Notably in this regard, expression of MUC1-C with mutation of the CQC motif to AQA blocks anchorage-independent growth and tumorigenicity of cancer cells, consistent with a dominant-negative effect [20, 22]. Based on those observations, cell-penetrating peptides, such as GO-203, were developed to target the MUC1-C CQC motif and inhibit MUC1-C-mediated survival mechanisms [23, 24]. Accordingly, targeting MUC1-C in cancer cells is achievable by several approaches that include (i) silencing with shRNAs, (ii) expression of the MUC1-C(CQC→AQA) mutant, and (iii) treatment with GO-203. Using these approaches, the present studies demonstrate that MUC1-C is of functional importance to KRAS dependency in NSCLC cells that harbor activating KRAS mutations. The results show that MUC1-C drives EMT and thereby confers stemness. Targeting MUC1-C thus reverses EMT and inhibits self-renewal in mutant KRAS NSCLC cells.

## RESULTS

### Silencing MUC1-C suppresses AKT in NSCLC cells with activating KRAS mutations

**Figure 1:**
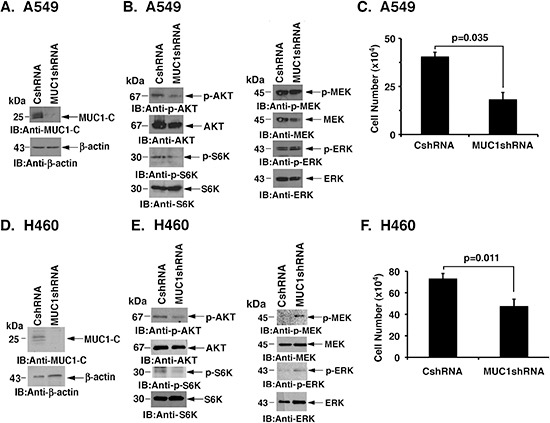
Silencing MUC1-C downregulates AKT and inhibits NSCLC cell growth **(A)** A549 cells were stably infected with lentiviruses expressing a control scrambled shRNA (CshRNA) or a MUC1 shRNA. Lysates were immunoblotted with the indicated antibodies. **(B)** Lysates from A549/CshRNA and A549/MUC1shRNA cells were immunoblotted with the indicated antibodies. **(C)** A549/CshRNA and A549/MUC1shRNA cells were plated at 5 × 10^4^ cells/well. The results (mean±SD of three replicates) are expressed as cell number on day 4. **(D)** H460 cells were stably infected with lentiviruses expressing CshRNA or MUC1shRNA. Lysates were immunoblotted with the indicated antibodies. **(E)** Lysates from H460/CshRNA and H460/MUC1shRNA cells were immunoblotted with the indicated antibodies. **(F)** H460/CshRNA and H460/MUC1shRNA cells were plated at 5 × 10^4^ cells/well. The results (mean±SD of three replicates) are expressed as cell number on day 4.

Human A549 NSCLC cells harbor the KRAS(G12S) mutation [25]. To assess the potential involvement of MUC1-C in activated KRAS signaling, A549 cells were infected with lentiviruses expressing a control CshRNA or one targeting MUC1-C (MUC1shRNA) (Fig. 1A). In A549/MUC1shRNA cells, we found that silencing MUC1-C results in decreased phosphorylation of AKT and the downstream effector S6K (Fig. 1B, left), but has no apparent effect on MEK and ERK activation (Fig. 1B, right). Silencing MUC1-C was also associated with a slowing of cell growth (Fig. 1C). To extend this analysis, H460/KRAS(Q61H) NSCLC cells were infected to stably express the CshRNA or MUC1shRNA (Fig. 1D). H460 cells similarly responded to downregulation of MUC1-C with suppression of AKT and S6K activation (Fig. 1E, left). Moreover, p-MEK and p-ERK levels were increased, consistent with a potential compensatory feedback response to decreases in AKT activity (Fig. 1E, right). As found for A549 cells, silencing MUC1-C resulted in inhibition of H460 cell growth (Fig. 1F).

### Targeting MUC1-C inhibits AKT and MEK signaling in KRAS mutant NSCLC cells

**Figure 2:**
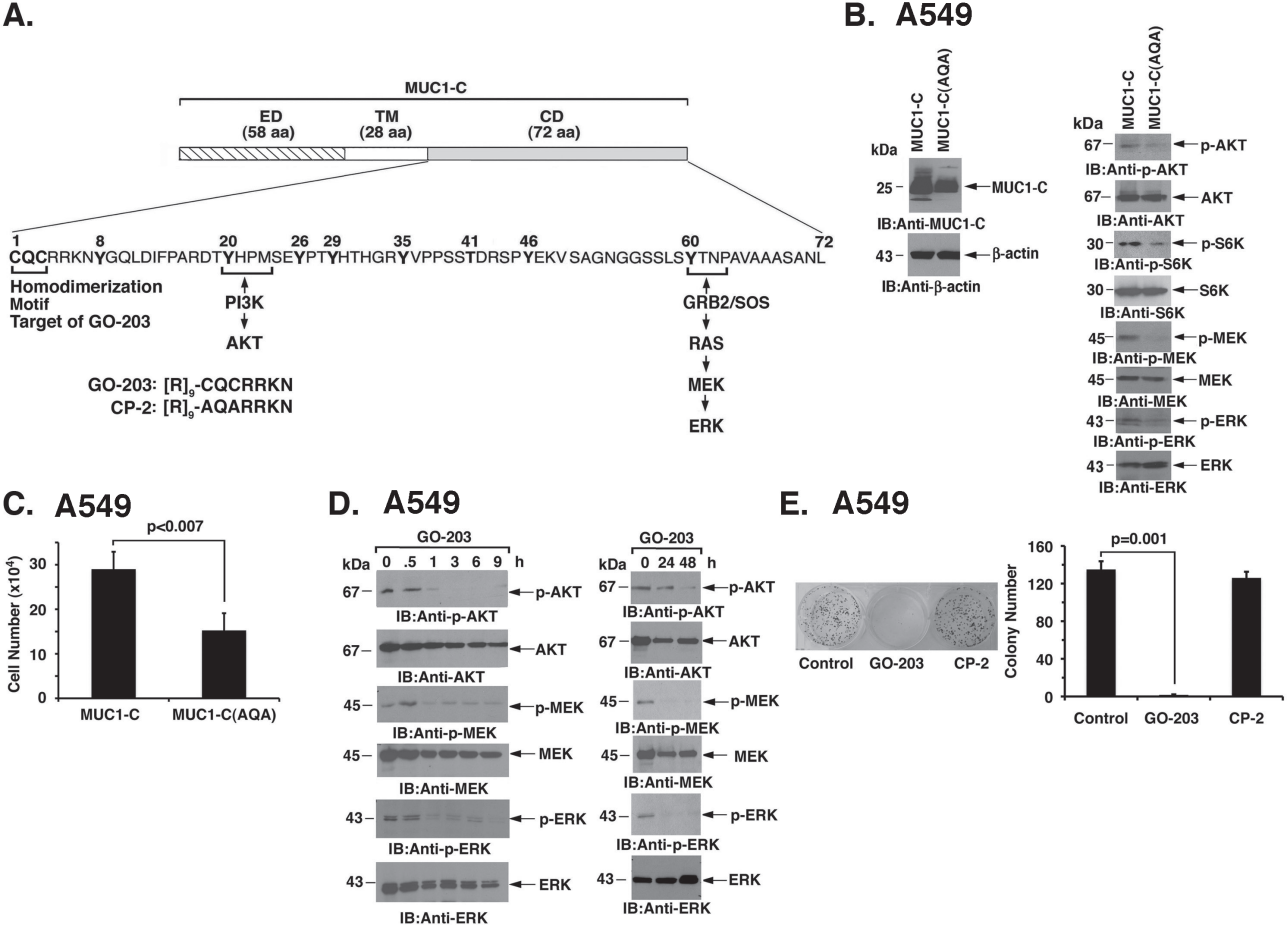
Targeting MUC1-C function suppresses AKT and MEK→ERK signaling **(A)** Schema of the MUC1-C subunit with the 58 aa extracellular domain (ED), 28 aa transmembrane domain (TM) and sequence of the 72 aa cytoplasmic domain (CD). The CQC motif is necessary for MUC1-C homodimerization and is the target for GO-203 treatment. Also highlighted are the binding sites that link the MUC1-C cytoplasmic domain to activation of the PI3K→AKT and MEK→ERK pathways. **(B)** A549 cells were stably transfected with vectors expressing MUC1-C or the MUC1-C(CQC→AQA) mutant [designated MUC1-C(AQA)]. Lysates were immunoblotted with the indicated antibodies. **(C)** A549/MUC1-C and A549/MUC1-C(AQA) cells were plated at 5 × 10^4^ cells/well. The results (mean±SD of three replicates) are expressed as cell number on day 4. **(D)** A549 cells were treated with 5 μM GO-203 at 0 and 24 h. Lysates were immunoblotted with the indicated antibodies (left and right). **(E)** A549 cells were seeded at 1000 cells/well in 6-well plates and left untreated (Control) or treated with 5 μM GO-203 or 5 μM CP-2 each day for 4 days. Colonies were stained with crystal violet on day 15 after treatment (left). Colony number (>30 cells) is expressed as the mean±SD of three replicates (right).

The MUC1-C subunit includes a 72 amino acid (aa) cytoplasmic domain with a CQC motif that is necessary and sufficient for MUC1-C homodimerization (Fig. 2A) [14]. Expression of MUC1-C with mutation of the CQC motif to AQA acts as a dominant-negative of MUC1-C function [22]. Accordingly, MUC1-C or MUC1-C(AQA) was stably overexpressed in A549 cells to assess the effects of the mutant (Fig. 2B, left). As found with MUC1-C silencing, overexpression of MUC1-C(AQA) was associated with suppression of AKT and S6K activation (Fig. 2B, right). In addition and in contrast to silencing MUC1-C, expression of MUC1-C(AQA) resulted in downregulation of MEK→ERK activation (Fig. 2B, right). MUC1-C(AQA) also inhibited A549 cell growth (Fig. 2C). The MUC1-C inhibitor, GO-203, is a cell penetrating peptide that contains a poly-Arg transduction domain linked to CQCRRKN (Fig. 2A). GO-203 blocks MUC1-C homodimerization and thereby its oncogenic function [21, 24]. Treatment of A549 cells with GO-203 was associated with transient downregulation of p-AKT levels at 3–9 h and then reactivation at 24 h (Fig. 2D, left and right). Retreatment with GO-203 at 24 h resulted in further suppression of AKT activation at 48 h (Fig. 2D, left and right). Moreover and like MUC1-C(AQA), we found that GO-203 inhibits MEK→ERK activation (Fig. 2D, left and right). GO-203 was also highly effective in inhibiting survival of A549 cells (Fig. 2E). By contrast, treatment with a control peptide CP-2 (Fig. 2A), that is inactive in inhibiting MUC1-C [21], had no effect on (i) AKT activity (Supplemental Fig. S1A) or (ii) loss of clonogenic survival (Fig. 2E). As confirmation of these findings, treatment of H460 cells with GO-203, but not CP-2 (Supplemental Fig. S1B), was similarly associated with suppression of AKT and MEK/ERK signaling (Supplemental Fig. S2A) and loss of survival (Supplemental Fig. S2B).

### Silencing MUC1-C suppresses ZEB1 expression

**Figure 3:**
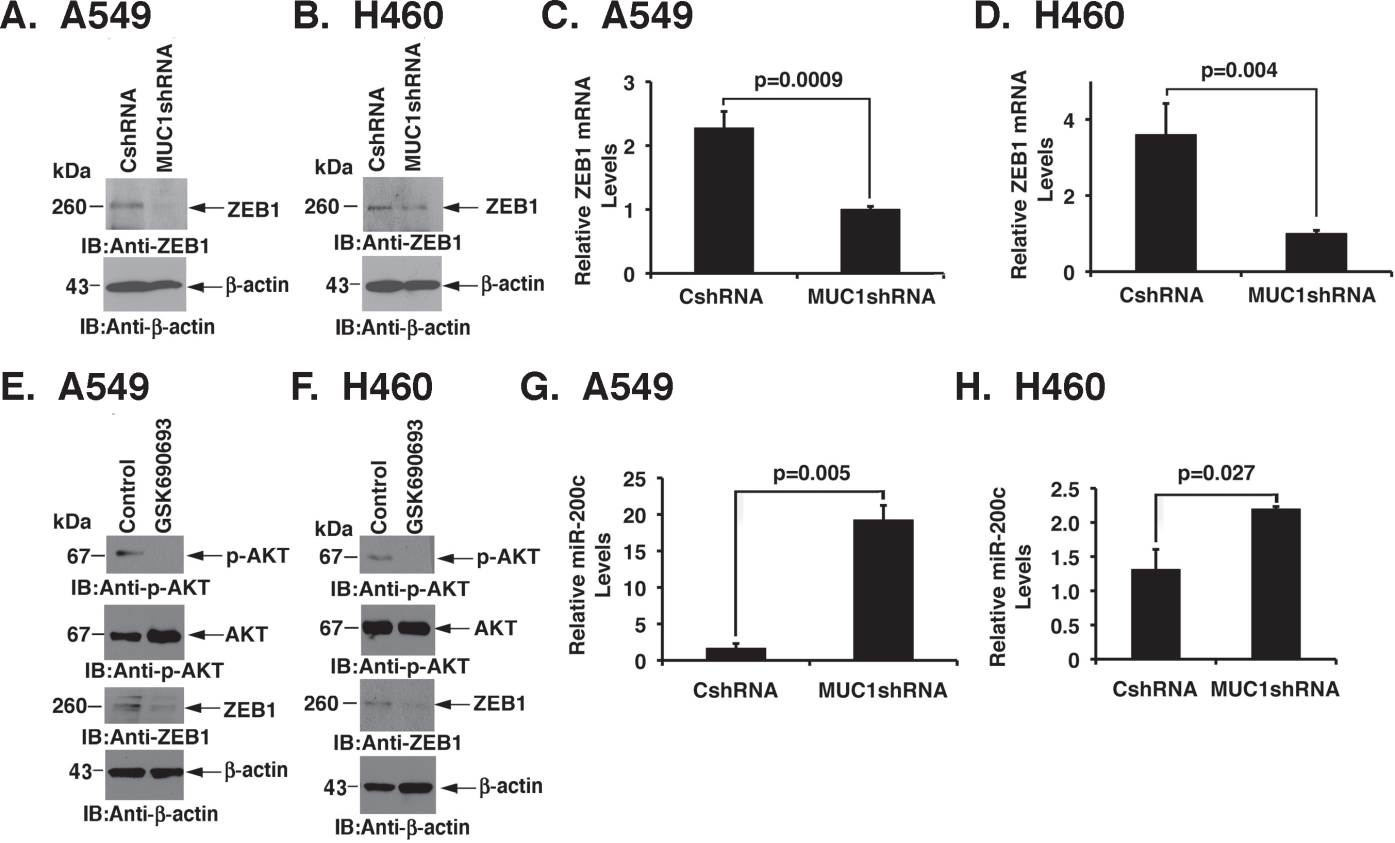
Silencing MUC1-C confers the coordinate downregulation of ZEB1 and induction of miR-200c expression **(A and B)** Lysates from A549 (A) and H460 (B) cells expressing CshRNA or MUC1shRNA were immunoblotted with the indicated antibodies. **(C and D)** ZEB1 mRNA levels for the indicated A549 (C) and H460 (D) cells were determined by qRT-PCR. The results are expressed as relative ZEB1 mRNA levels (mean±SD of three determinations) as compared to that obtained for GAPDH as a control. **(E and F)** A549 (E) and H460 (F) cells were left untreated or treated with 10 μM GSK690693 for 48 h. Lysates were immunoblotted with the indicated antibodies. **(G and H)** Relative miR-200c levels in the indicated A549 (G) and H460 (H) cells were determined by qRT-PCR. The results are expressed as relative miR-200c levels (mean±SD of three determinations) as compared to that obtained for U6 as a control.

A549 and H460 cells exhibit an EMT phenotype, which is a characteristic of importance for KRAS independence [7]. To determine if MUC1-C regulates EMT in these NSCLC cells, we first studied the effects of silencing MUC1-C on expression of the ZEB1 transcription factor and inducer of the mesenchymal phenotype [26]. Downregulation of MUC1-C in A549 cells was associated with marked suppression of ZEB1 levels (Fig. 3A). In H460 cells, ZEB1 expression was also decreased in response to MUC1-C silencing (Fig. 3B). In addition, we found that silencing MUC1-C suppresses ZEB1 mRNA levels in A549 and H460 cells (Figs. 3C and D), consistent with involvement of MUC1-C in upregulating *ZEB1* transcription. In contrast to the KRAS-independent A549 and H460 cells and consistent with previous observations [7], there was no detectable ZEB1 expression in the KRAS-dependent H358 and H441 cells (data now shown). Activation of AKT has been linked to the induction of ZEB1 expression [27, 28]. In concert with those observations and the demonstration that targeting MUC1-C suppresses AKT and ZEB1, we found that inhibiting AKT with GSK690693 is associated with downregulation of ZEB1 in A549 and H460 cells (Figs. 3E and F). Moreover and consistent with ZEB1-mediated suppression of miR-200c [26], we found that silencing MUC1-C is associated with induction of miR-200c levels (Figs. 3G and H). These findings provided support for a model in which MUC1-C contributes to the activation of AKT and thereby the coordinate induction of ZEB1 and suppression of miR-200c expression.

### Silencing MUC1-C reverses EMT and KRAS independence

**Figure 4:**
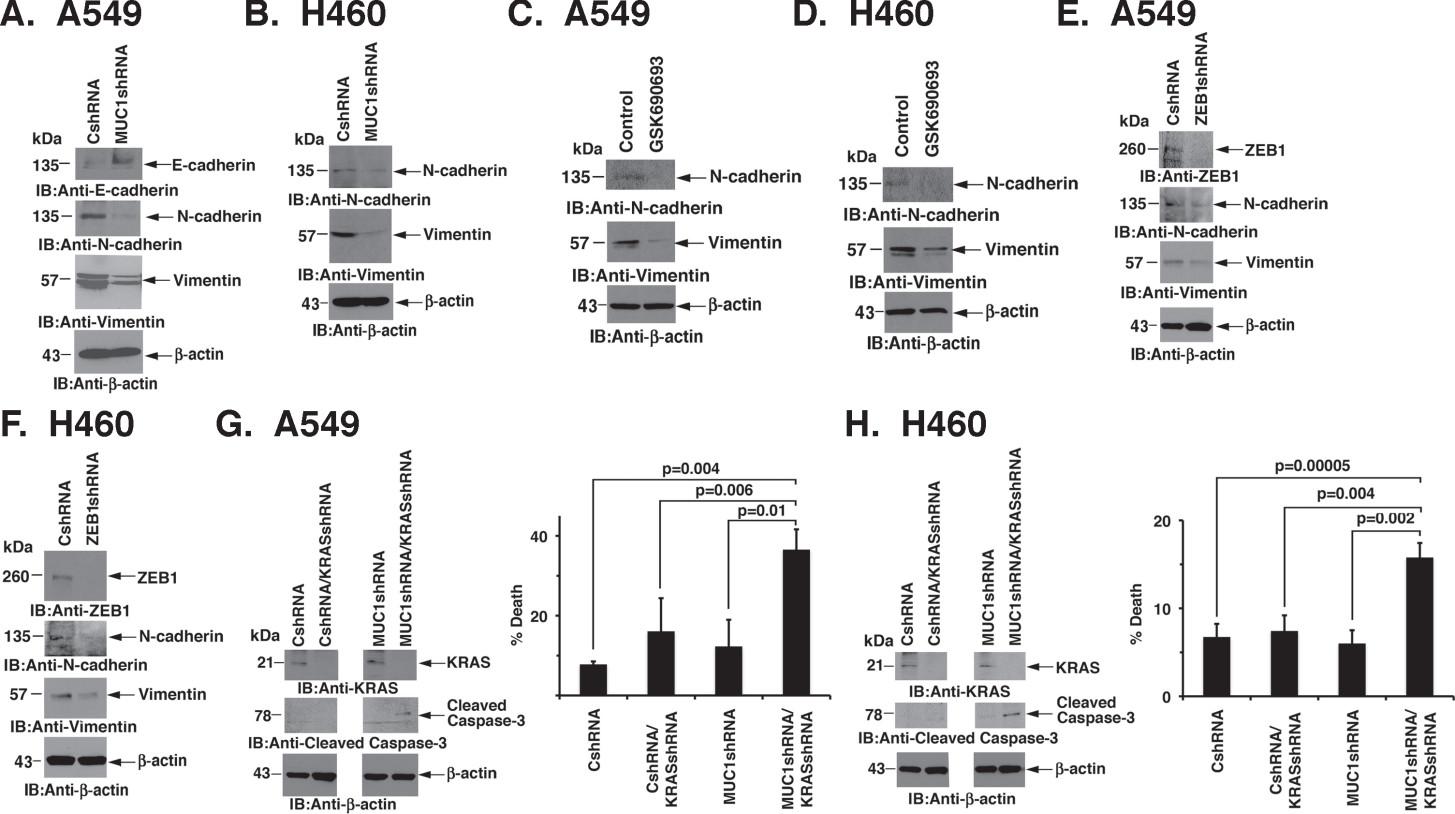
Silencing MUC1-C reverses EMT and KRAS independence **(A and B)** Lysates from A549 (A) and H460 (B) cells expressing CshRNA or MUC1shRNA were immunoblotted with the indicated antibodies. **(C and D)** A549 and H460 cells were left untreated or treated with 10 μM GSK690693 for 48 h. Lysates were immunoblotted with the indicated antibodies. **(E and F)** Lysates from A549 (E) and H460 (F) cells expressing CshRNA or MUC1shRNA were immunoblotted with the indicated antibodies. **(G and H)** A549/CshRNA and A549/MUC1shRNA (G) or H460/CshRNA and H460/MUC1shRNA (H) cells were infected twice over 24 h with lentivirus expressing a KRAS shRNA. At 48 h post-infection, cells were (i) collected for immunoblotting with the indicated antibodies (left) or (ii) plated at a density of 5 × 10^4^ in a 6-well plate. The results (mean±SD of three replicates) are expressed as percent cell death as determined by trypan blue exclusion on day 4 (right).

miR-200c is an inducer of epithelial differentiation [26]. Thus, with the suppression of ZEB1 and induction of miR-200c, silencing MUC1-C in A549 cells was associated with upregulation of E-cadherin, and decreases in N-cadherin and vimentin, consistent with reversal of EMT (Fig. 4A). In H460 cells, E-cadherin was not detectable in the absence or presence of MUC1-C silencing. However, downregulation of MUC1-C resulted in decreased expression of N-cadherin and vimentin (Fig. 4B). Similar results were obtained when A549 and H460 cells were treated with the AKT inhibitor, linking suppression of AKT to the reversal of EMT (Figs. 4C and D). In addition, to confirm that the downregulation of ZEB1 in response to MUC1-C silencing is also responsible for reversing EMT, we silenced ZEB1 and found induction of the mesenchymal-epithelial transition (MET) as evidenced by decreases in N-cadherin and vimentin (Figs. 4E and F). EMT has been linked to KRAS independence in mutant KRAS NSCLC cells [7]. Accordingly, we asked if silencing MUC1-C converts KRAS independence to dependence on KRAS for survival. Indeed, the downregulation of KRAS in A549/MUC1shRNA cells was associated with increases in caspase-3 cleavage (Fig. 4G, left) and cell death (Fig. 4G, right) as compared to that obtained for A549/CshRNA cells. Similar results were obtained in studies of H460/CshRNA and H460/MUC1shRNA cells with suppression of KRAS expression (Fig. 4H, left and right), indicating that MUC1-C contributes to KRAS independence.

### Targeting MUC1-C function induces MET

**Figure 5:**
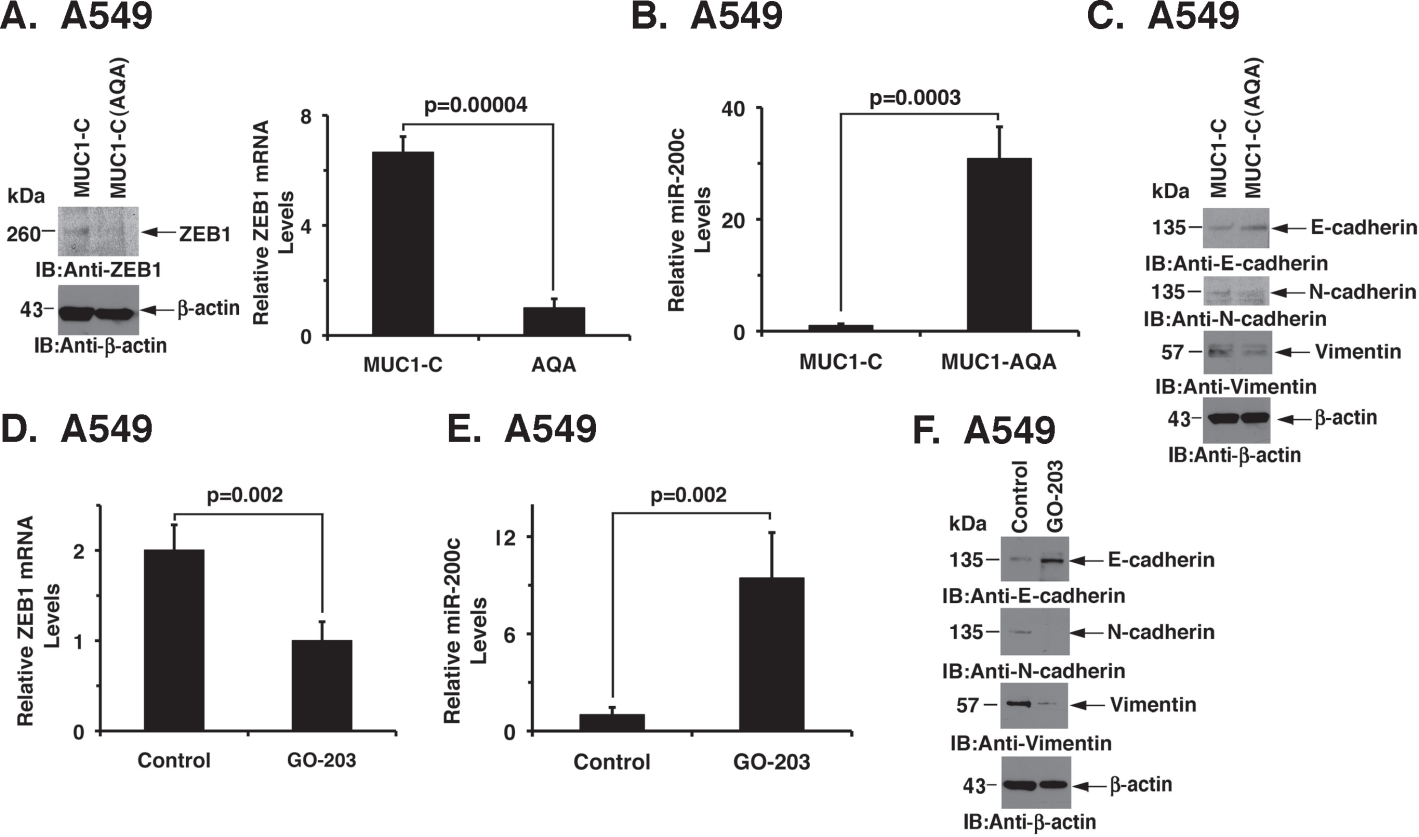
Targeting MUC1-C induces MET **(A)** Lysates from A549/MUC1-C and A549/MUC1-C(AQA) cells were immunoblotted with the indicated antibodies (left). ZEB1 mRNA levels were determined by qRT-PCR. The results are expressed as relative ZEB1 mRNA levels (mean±SD of three determinations) as compared to that obtained for GAPDH as a control (right). **(B)** miR-200c levels in A549/MUC1-C and A549/MUC1-C(AQA) cells were determined by qRT-PCR. The results are expressed as relative miR-200c levels (mean±SD of three determinations) as compared to that obtained for U6 as a control. **(C)** Lysates from A549/MUC1-C and A549/MUC1-C(AQA) were immunoblotted with the indicated antibodies. **(D)** A549 cells were left untreated or treated with 5 μM GO-203 for 48 h. ZEB1 mRNA levels were determined by qRT-PCR. The results are expressed as relative ZEB1 mRNA levels (mean±SD of three determinations) as compared to that obtained for GAPDH as a control. **(E)** A549 cells were left untreated or treated with 5 μM GO-203 for 48 h. Relative miR-200c levels were determined by qRT-PCR. The results are expressed as relative miR-200c levels (mean±SD of three determinations) as compared to that obtained for U6 as a control. **(F)** A549 cells were left untreated or treated with 5 μM GO-203 for 48 h. Lysates were immunoblotted with the indicated antibodies.

As noted above and in addition to MUC1-C silencing, we studied the effects of targeting MUC1-C function on EMT by (i) stable expression of the MUC1-C(AQA) mutant, and (ii) treatment with the MUC1-C inhibitor GO-203. Overexpression of MUC1-C(AQA) in A549 cells was associated with decreases in ZEB1 (Fig. 5A, left and right) and upregulation of miR-200c expression (Fig. 5B). In addition, the A549/MUC1-C(AQA) cells exhibited reversal of EMT, as evidenced by increases in E-cadherin and suppression of N-cadherin and vimentin (Fig. 5C). Treatment of A549 cells with GO-203 also decreased ZEB1 mRNA and increased miR-200c levels (Figs. 5D and E). Moreover, GO-203 treatment was associated with the acquisition of an epithelial phenotype (Fig. 5F). Thus, inhibition of MUC1-C function with different approaches supported the notion that MUC1-C drives EMT in mutant KRAS NSCLC cells.

### MUC1-C promotes self-renewal

**Figure 6:**
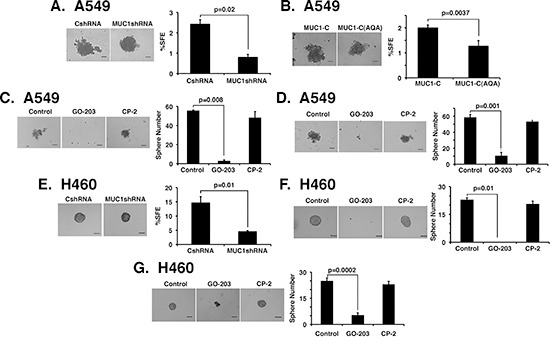
MUC1-C is necessary for self-renewal **(A and B)** Representative images are shown for the indicated A549 cells plated at 2000 cells/well and grown for 5 days in sphere culture (left). Bar represents 100 microns. The percentage SFE is expressed as the mean±SD of three determinations (right). **(C)** A549 cells were plated at 2000 cells/well in sphere culture and left untreated (Control) or treated with 5 μM GO-203 or CP-2 for 3 days. Representative images on day 5 are shown for the indicated A549 cells (left). The percentage SFE is expressed as the mean±SD of three determinations (right). **(D)** A549 cells were plated at 2000 cells/well and cultured for 5 days. The established spheres were then left untreated (Control) or treated with 5 μM GO-203 or CP-2 for 3 days. Representative images are shown for the indicated A549 cells (left). The percentage SFE is expressed as the mean±SD of three determinations (right). **(E)** Representative images are shown for the indicated H460 cells plated at 1500 cells/well and grown for 5 days in sphere culture (left). Bar represents 100 microns. The percentage SFE is expressed as the mean±SD of three determinations (right). **(F)** H460 cells were plated at 1500 cells/well in sphere culture and left untreated (Control) or treated with 5 μM GO-203 or CP-2 for 3 days. Representative images on day 5 are shown for the indicated H460 cells (left). The percentage SFE is expressed as the mean±SD of three determinations (right). **(G)** H460 cells were plated at 1500 cells/well and cultured for 5 days. The established spheres were then left untreated (Control) or treated with 5 μM GO-203 or CP-2 for 3 days. Representative images are shown for the indicated H460 cells (left). The percentage SFE is expressed as the mean±SD of three determinations (right).

Sphere formation under non-adherent growth conditions selects for the expansion of self-renewing cancer stem-like cells (CSCs), which survive anoikis [29, 30]. Silencing MUC1-C had little effect on the size of A549 spheres (Fig. 6A, left). However, downregulation of MUC1-C expression significantly decreased the sphere forming efficiency (%SFE) of A549 cells (Fig. 6A, right). Similar results were obtained when studying A549 cells expressing the MUC1-C(AQA) mutant (Fig. 6B). Treatment of A549 cells with GO-203, but not CP-2, blocked sphere formation (Fig. 6C, left and right). Moreover, when A549 spheres were established and then treated with peptide, GO-203 was effective in disrupting the spheres, whereas CP-2 had little if any effect (Fig. 6D, left and right). In studies of H460 cells, we also found that (i) MUC1-C silencing (Fig. 6E, left and right) and (ii) GO-203 treatment (Fig. 6F, left and right) are associated with suppression of sphere formation. In addition, GO-203 was effective in conferring the disruption of established H460 spheres (Fig. 6G, left and right). These results provided support for the involvement of MUC1-C in self-renewal of A549 and H460 cells.

### KRAS mutant NSCLC tumorigenicity is MUC1-C-dependent

**Figure 7:**
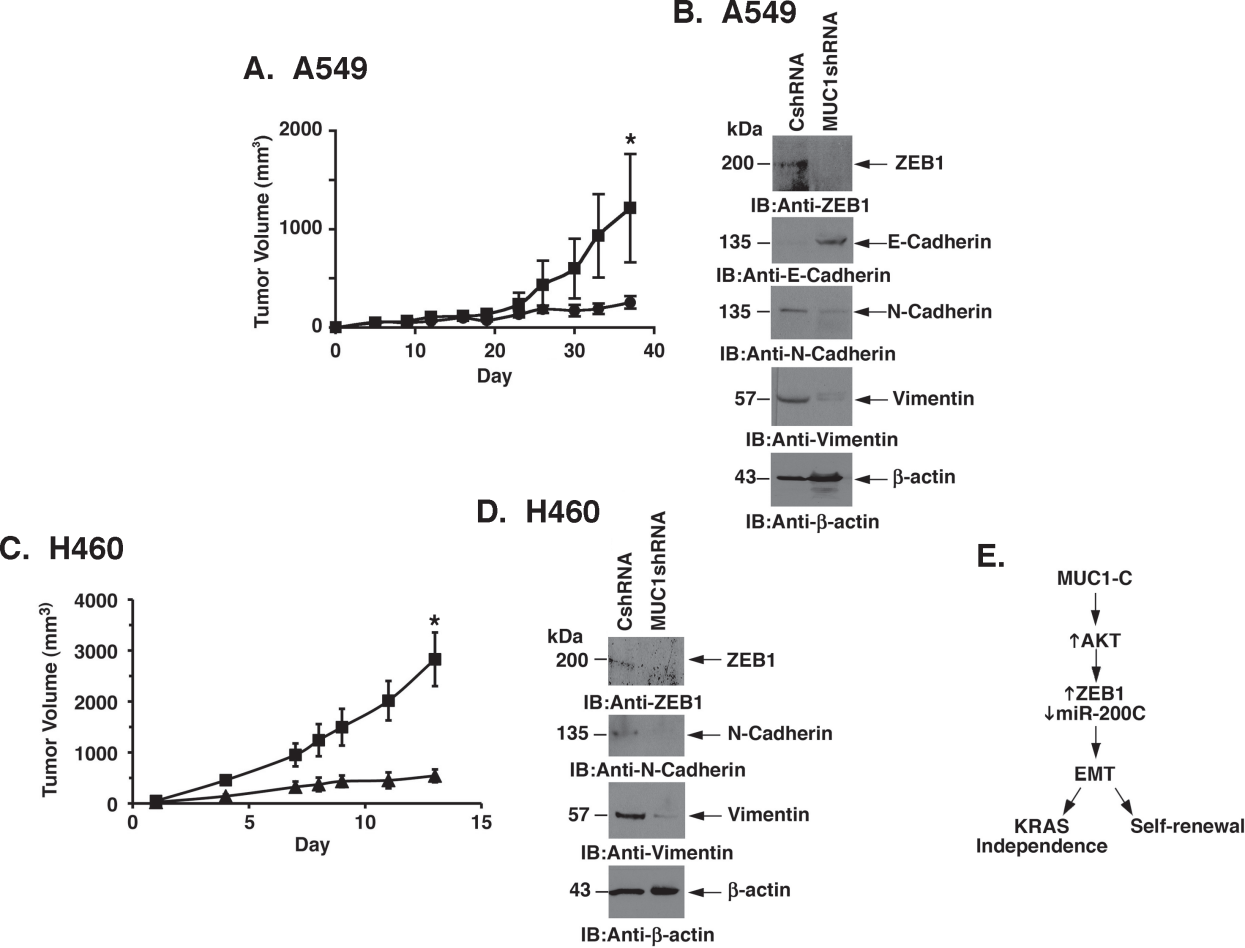
MUC1-C promotes mutant KRAS NSCLC cell tumorigenicity **(A)** A549/CshRNA (squares) and A549/ MUC1shRNA (circles) cells (4 × 10^6^) were injected subcutaneously in the flanks of female nude mice. Tumor volumes were determined on the indicated days after injection. The results are expressed as tumor volumes (mean±SEM for 3 mice). The asterisk denotes a significant difference (p=0.02) between growth of the A549/CshRNA and A549/MUC1shRNA tumors on day 38. **(B)** Lysates from tumors isolated on day 30 from mice in the different treatment groups were immunoblotted with indicated antibodies. **(C)** H460/CshRNA (squares) and H460/MUC1shRNA (triangles) cells (4 × 10^6^) were injected subcutaneously in the flanks of female nude mice. Tumor volumes were determined on the indicated days after injection. The results are expressed as tumor volumes (mean±SEM for 3 mice). The asterisk denotes a significant difference (p=0.013) between growth of the H460/CshRNA and H460/MUC1shRNA tumors on day 13. **(D)** Lysates from tumors isolated on day 13 from mice in the different treatment groups were immunoblotted with indicated antibodies. **(E)** Schema depicting the proposed pathway in which MUC1-C activates AKT and thereby the coordinate induction of ZEB1 and suppression of miR-200c. In turn, MUC1-C drives EMT, self-renewal and KRAS independence.

To extend the findings that MUC1-C promotes self-renewal, we investigated the effects of silencing MUC1-C on tumorigenicity of A549 cells. Consistent with the decreased capacity for sphere formation, growth of A549/MUC1shRNA cells was significantly inhibited as compared to that found for A549/CshRNA cells (Fig. 7A). As found in in vitro studies, immunoblot analysis of the A549 tumors further showed that MUC1-C silencing is associated with downregulation of ZEB1, increases in E-cadherin, and decreases in N-cadherin and vimentin (Fig. 7B). Growth of H460 cells as tumor xenografts was also slowed as a consequence of MUC1-C silencing (Fig. 7C). Moreover, the H460/MUC1shRNA tumors exhibited decreases in ZEB1, N-cadherin and vimentin, consistent with the MET phenotype (Fig. 7D).

## DISCUSSION

Targeting mutant KRAS in NSCLC and other types of tumors with small molecule inhibitors has been unsuccessful to date [1]. Therapeutic approaches have therefore focused on the downstream AKT and MEK pathways which can confer dependence on mutant KRAS for survival [1]. The MUC1-C oncoprotein is aberrantly expressed in NSCLC and is associated with poor clinical outcomes [12, 13]; however, little was known about whether MUC1-C contributes to mutant KRAS signaling. To address this issue, we used three strategies to inhibit MUC1-C function and thereby assess effects on the KRAS downstream AKT and MEK pathways. Consistent with a direct interaction between the MUC1-C cytoplasmic domain and PI3K [24, 31], we found that silencing MUC1-C NSCLC cells harboring mutant KRAS is associated with downregulation of the AKT pathway, but has no apparent effect on MEK→ERK signaling. The MUC1-C oncogenic function is dependent on the formation of MUC1-C homodimers through a CQC motif in the cytoplasmic domain [14, 32]. Interestingly and as a second approach, stable expression of a MUC1-C(CQC→AQA) mutant, which acts as a dominant-negative of MUC1-C function [22], resulted in suppression of both AKT and MEK. As a third approach, treatment with the GO-203 inhibitor, which binds to the MUC1-C CQC site and blocks MUC1-C homodimerization, also suppressed both AKT and MEK activity. In contrast to targeting the MUC1-C CQC motif with the MUC1-C(AQA) mutant or GO-203, the absence of MEK downregulation in response to stable MUC1-C silencing may represent a compensatory response to suppression of AKT, as has been observed in other settings [33]. Nonetheless, our results demonstrate that inhibiting MUC1-C in several different ways is associated with suppression of key pathways that reside downstream of mutant KRAS and are necessary for growth and survival.

Concurrent inhibition of the AKT and MEK pathways in a murine lung adenocarcinoma model driven by mutant *Kras* is associated with significant tumor regressions [2]. The precise mechanisms responsible for mutant KRAS cell death in response to inhibiting AKT and MEK signaling remain unclear; however, several studies indicate that these pathways converge in the regulation of MYC expression [34]. In this way, MYC has been shown to be of importance for the survival of cells with activated KRAS [35]. The present studies do not exclude the possibility that MUC1-C can contribute to the regulation of MYC in mutant KRAS NSCLC cells and therefore further studies will be needed to address this possibility. From a mechanistic standpoint, NSCLC cells with activated KRAS can vary in their dependency on KRAS [7, 36]. Engagement of the AKT and MEK pathways, and sensitivity to their respective inhibitors, are not a function of KRAS dependency [7]. Rather, extent of KRAS dependency appears to reside in downstream regulation of EMT [7]. Notably in this regard, recent studies have shown that MUC1-C induces EMT in breast cancer cells by the upregulation of ZEB1 and the coordinate suppression of miR-200c [17]. In the present studies, silencing MUC1-C in NSCLC cells decreased ZEB1, which in turn was associated with increases in miR-200c, and significantly, reversal of the EMT phenotype. The demonstration that targeting MUC1-C with the MUC1-C(AQA) mutant or GO-203 resulted in similar responses, providing support for the notion that MUC1-C is necessary for driving ZEB1 and thereby EMT in these NSCLC cells. AKT has been shown to contribute to the induction of ZEB1 expression [27, 28]. In concert with the involvement of AKT, we also found that inhibition of AKT suppressed ZEB1 expression. These findings thus support a model in which targeting MUC1-C downregulates AKT and thereby ZEB1 expression by an AKT-mediated mechanism (Fig. 7E).

The coordinate upregulation of ZEB1 and suppression of miR-200c, an inducer of epithelial differentiation, is associated with the induction of EMT [26]. Accordingly, we found that targeting MUC1-C with the downregulation of ZEB1 and induction of miR-200c resulted in MET, indicating that MUC1-C is necessary for conferring the EMT phenotype in these KRAS-independent NSCLC cells (Fig. 7E). Notably, EMT dictates the dependency of NSCLC cells on activated KRAS [7]. By extension, silencing MUC1-C with reversal of EMT was associated with an increase in the KRAS dependency index. Similar enhancement of KRAS dependency was observed when targeting MUC1-C with the MUC1-C(AQA) mutant and GO-203, indicating that MUC1-C dictates EMT and KRAS dependency (Fig. 7E). EMT has been linked to cancer stem-like cells that have been characterized by the endowment of mesenchymal traits necessary for invasion and metastases [37]. In this way, EMT promotes the capacity to form spheres in non-adherent serum-free culture, a characteristic that is dependent on the presence of self-renewing stem-like cells [29, 30]. Therefore, the finding that MUC1-C confers the EMT phenotype in KRAS mutant NSCLC cells invoked the possibility that this observation could extend to self-renewal (Fig. 7E). Indeed, we found that targeting MUC1-C with silencing or expression of the MUC1-C(CQC→AQA) mutant decreased sphere forming efficiency. Disruption of established spheres with the GO-203 inhibitor further demonstrated that these KRAS mutant stem-like cells are dependent on MUC1-C for their self-renewal. The involvement of MUC1-C in the capacity for self-renewal is further supported by the demonstration that silencing MUC1-C substantially decreases tumorigenicity of KRAS mutant NSCLC cells growing in nude mice. The available evidence indicates that cancer stem-like cells maintain low levels of reactive oxygen species (ROS) and that disruption of ROS defense mechanisms results in loss of self-renewal [38–41]. In this context, MUC1-C protects cells from increases in ROS associated with exposure to oxidative stress, hypoxia and glucose deprivation [32]. These observations and the sensitivity of cancer stem-like cells to disruption of redox balance lend credence to the possibility that MUC1-C is necessary for maintenance of ROS levels and thereby self-renewal.

Finally, the present work has focused on MUC1-C function in mutant KRAS-independent NSCLC cells that express ZEB1 and exhibit the EMT phenotype. Further studies will be needed to assess the role of MUC1-C in mutant KRAS-dependent NSCLC cells that are ZEB1 negative and exhibit epithelial characteristics. Nonetheless, the present findings indicate that targeting MUC1-C could be an effective therapeutic approach for at least certain mutant KRAS NSCLCs. In this respect, GO-203 has completed Phase I evaluation in patients with refractory solid tumors and a maximum tolerated dose has been identified for Phase II trials. Given the challenges being encountered in the treatment of mutant KRAS NSCLCs, our results lend support to the notion that targeting MUC1-C with GO-203 could be an alternative approach for these patients.

## METHODS

### Cell culture

Human A549/KRAS(G12S), H460/KRAS(Q61H), H358/KRAS(G12C) and H441/KRAS(G12V) NSCLC cells (ATCC) were grown in RPMI1640 media supplemented with 10% heat-inactivated fetal bovine serum (HI-FBS), 100 μg/ml streptomycin, 100 units/ml penicillin and 2 mM L-glutamine. Authenticity of the cells was confirmed by short tandem repeat (STR) DNA profiling (Dana-Farber Cancer Institute, Molecular Biology Core). Cells were infected with lentiviral vectors expressing a MUC1 shRNA (Sigma), a scrambled control shRNA (CshRNA; Sigma) or a KRAS shRNA (Sigma). Cells were also transfected to stably express a control pHR-CMV vector expressing MUC1-C or one expressing MUC1-C(CQC→AQA). Cells were treated with the MUC1-C inhibitor peptide GO-203, a control peptide CP-2 [24] or with the AKT inhibitor GSK690693 (Selleck Chemicals).

### Immunoblot analysis

Cell lysates were prepared as described [23]. The lysates were analyzed by immunoblotting with anti-MUC1-C [42], anti-p-AKT, anti-AKT, anti-p-S6K, anti-S6K, anti-p-MEK(Ser-217/221), anti-MEK, anti-p-ERK(Thr-202/Tyr-204), anti-ERK (Cell Signaling Technologies), anti-β-actin (Sigma), anti-KRAS (Santa Cruz Biotechnology) or anti-caspase-3 (Cell Signaling Technologies) as described [24, 43]. Immune complexes were detected with horseradish peroxidase-conjugated secondary antibodies and enhanced chemiluminescence (GE Healthcare).

### Colony formation assays

Cells were seeded in 6-well plates for 24 h and then left untreated or treated with inhibitor. After 7–14 d, the cells were washed and stained with 0.5% crystal violet in 25% methanol. Colonies >30 cells were counted in triplicate wells.

### Quantitative RT-PCR

cDNA synthesis was performed with 1 μg of total RNA using the Thermoscript RT-PCR assay system (Invitrogen). The cDNA samples were diluted and amplified using the SYBR green qPCR assay kit (Applied Biosystems) and the ABI Prism 7000 Sequence Detector (Applied Biosystems). Primers used for ZEB1 and glyceraldehyde 3-phosphate dehydrogenase have been previously reported [17].

### Analysis of miR-200c

RNA was isolated using the miRNeasy kit (Qiagen). cDNAs were prepared from 1 μg RNA using the cDNA synthesis for small RNAs (System Biosciences). miR-200c was detected using a specific forward primer and a universal reverse primer as described [17]. Human U6 small RNA was used as a control [17]. The SYBR green qPCR assay kit (Applied Biosystems) was used with 1 μl of diluted cDNA sample and analyzed with the ABI Prism 7000 Sequence Detector (Applied Biosystems).

### Tumor spheres

NSCLC cells were harvested with gentle trypsinization, washed and resuspended in MammoCult^™^ Human Medium (Stem Cell Technologies). Single cells were confirmed under a microscope, counted, seeded in 6-well ultralow attachment culture plates (Corning CoStar) and cultured for 5 days. Tumor spheres of ≥100 μm were visualized and scored using a Nikon inverted TE2000 microscope. Sphere forming efficiency (SFE) was calculated by dividing the number of tumor spheres by the number of suspended cells.

### NSCLC xenograft models

Four- to 6-week old BALB/c nu/nu mice were injected subcutaneously with 4 × 10^6^ cells in the flank. Tumor volumes were calculated using the formula V=L^2^ × W/2, where L and W are the larger and smaller diameters, respectively.

## SUPPLEMENTARY FIGURES



## Abbreviations

NSCLC: non-small cell lung cancer
MUC1: mucin 1
MUC1-C: MUC1 C-terminal subunit
EMT: epithelial-mesenchymal transition
MET: mesenchymal-epithelial transition
