# Supplementary material for: MUC1-C confers EMT and KRAS independence in mutant KRAS lung cancer cells
Source: Oncotarget. 2014 Aug 22;5(19):8893–905. doi: 10.18632/oncotarget.2360 (PMC4253405; doi:10.18632/oncotarget.2360)
Supplement: Supplementary file 1 [file oncotarget-05-8893-s001.pdf]

SUPPLEMENTARY FIGURES

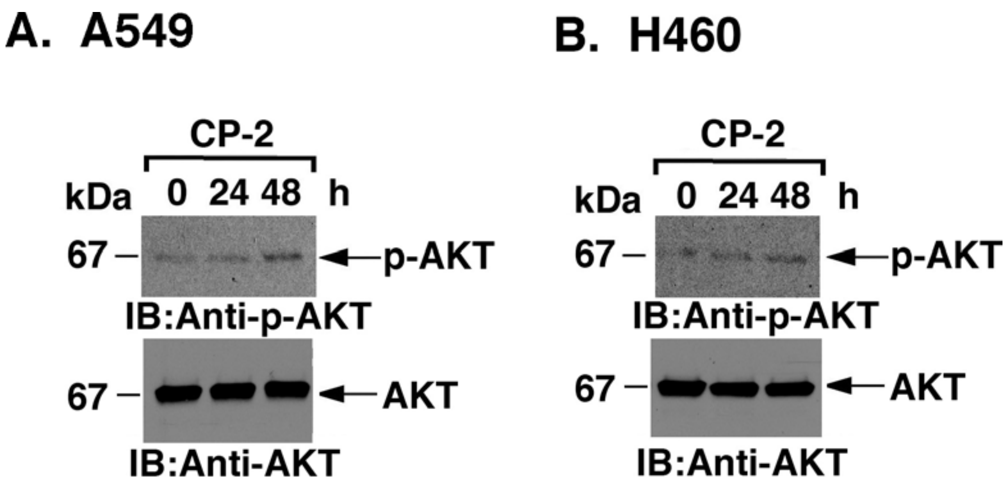

Supplementary Figure S1: (A) A549 cells were treated with 5 µM CP-2 at 0 and 24 h. Lysates were immunoblotted with the indicated antibodies. **(B)** H460 cells were treated with 5 µM CP-2 at 0 and 24 h. Lysates were immunoblotted with the indicated antibodies.

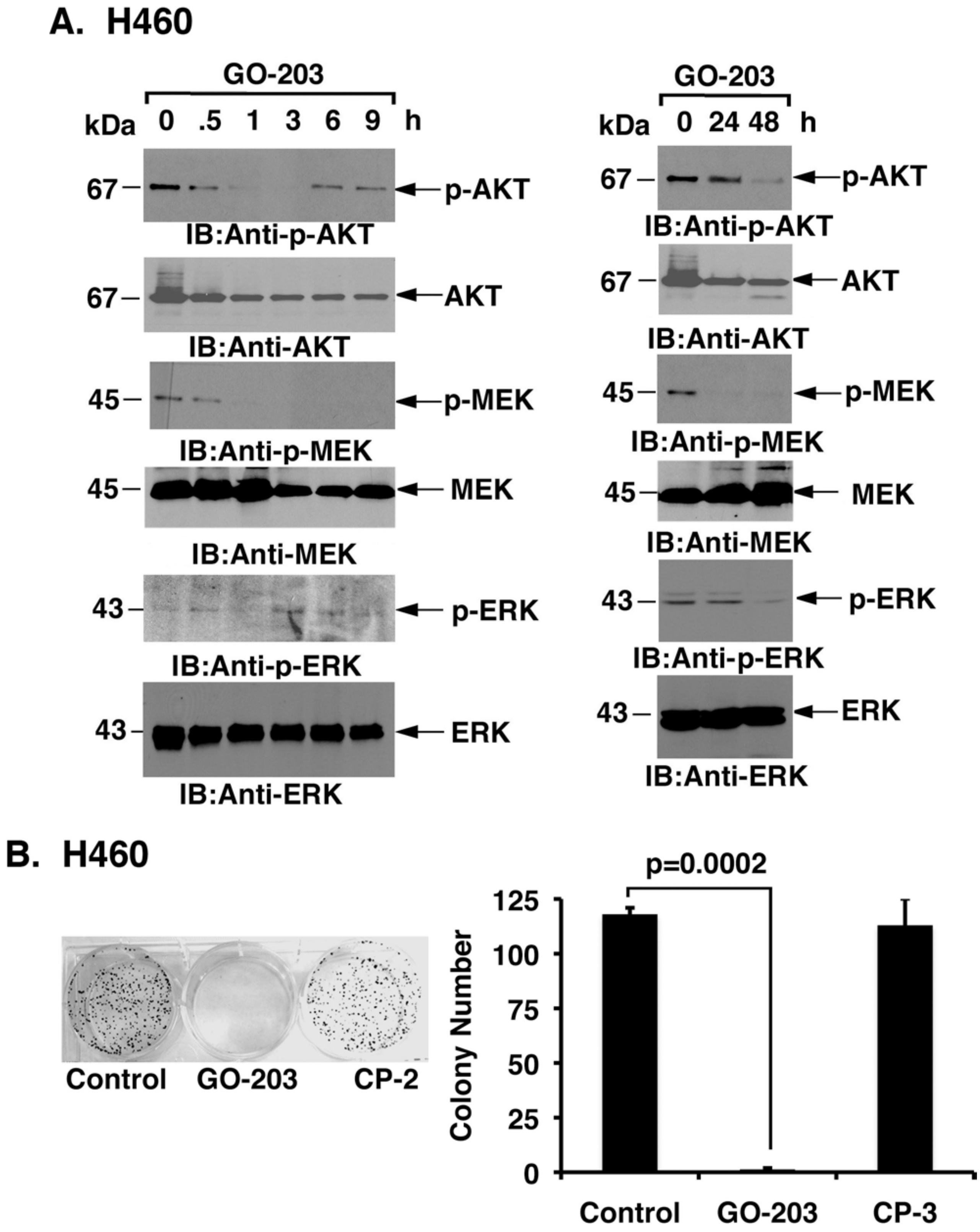

Supplementary Figure S2: (A) H460 cells were treated with 5 μM GO-203 at 0 and 24 h. Lysates were immunoblotted with the indicated antibodies (left and right). **(B)** H460 cells were seeded at 500 cells/well in 6-well plates and left untreated (Control) or treated with 5 μM GO-203 or CP-2 each day for 4 days. Colonies were stained with crystal violet on day 15 after treatment (left). Colony number (>30 cells) is expressed as the mean±SD of three replicates (right).
